# Improvement in left atrial strain after percutaneous mitral valvuloplasty is an independent prognostic marker in patients with rheumatic mitral stenosis

**DOI:** 10.3389/fcvm.2026.1719283

**Published:** 2026-05-15

**Authors:** Alexandre Negrao Pantaleao, Fernanda Azevedo Figueiredo, Antonio Mutarelli, Bruna Vieira Guarçoni, Hortência Carvalho, Isabella Fonseca, Shani Dahan, Jagdip Kang, Rasmus Sindre, Juliana Rodrigues Soares Oliveira, Elena Aikawa, Robert A. Levine, Maria Carmo Pereira Nunes, on behalf of the PRIMA Network

**Affiliations:** 1 School of Medicine, Universidade Federal de Minas Gerais, Belo Horizonte, Minas Gerais, Brazil; 2 Cardiac Ultrasound Laboratory, Massachusetts General Hospital, Harvard Medical School, Boston, MA, United States; 3 Department of Clinical Sciences, University of Bergen, Bergen, Norway; 4 Center for Interdisciplinary Cardiovascular Sciences and Center for Excellence in Vascular Biology, Cardiovascular Medicine, Brigham and Women’s Hospital, Harvard Medical School, Boston, MA, United States

**Keywords:** echocardiogaphy, left atrial reservoir strain, mitral stenosis (MS), rheumatic fever (RF), rheumatic heart disease (RHD)

## Abstract

**Background:**

Rheumatic mitral stenosis (MS) leads to left atrial (LA) pressure overload, resulting in progressive atrial remodeling and dysfunction. Percutaneous mitral valvuloplasty (PMV) relieves LA pressure overload and is the primary treatment for severe MS. LA reservoir strain (LASr), assessed by speckle-tracking echocardiography, allows early detection of LA dysfunction, which may improve immediately after the procedure, with potential prognostic implications. In this study, we analyzed the prognostic value of post-procedural improvement in LASr beyond well-established predictors.

**Methods:**

Patients with rheumatic MS undergoing PMV were prospectively recruited. All patients underwent a transthoracic echocardiogram before and within 48 h after the procedure. The primary outcome was a composite of cardiac death or need for mitral valve replacement (MVR). The prognostic value of the change in LASr post and pre PMV (ΔLASr) was assessed using a Cox proportional hazards model adjusted for known prognostic factors.

**Results:**

In total, 163 patients were included. Overall, patients were aged 46 (IQR 37–54) years, 82.8% were female, median mitral valve area pre PMV was 0.97 (IQR 0.82–1.13) cm^2^ and median ΔLASr was +3.2 (IQR −0.4 to +6.2). Over a median follow-up of 55 months (IQR 27–83), 46 patients had the composite outcome (28%), being 9 cardiac deaths and 37 MVR. In the final Cox model, ΔLASr was an independent predictor of the composite outcome [HR: 0.93 (0.87–0.99), *p* = 0.021].

**Conclusion:**

Improvement in LASr independently predicted clinical outcomes in patients with rheumatic MS undergoing PMV, with each unit increase in ΔLASr associated with a 7% risk reduction of death or MVR. Assessment of LASr may be a valuable tool for post-procedural risk stratification in this population.

## Graphical Abstract

**Figure:**
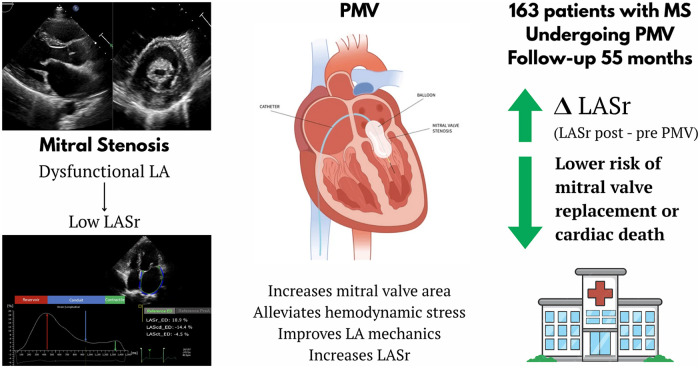

## 1 Introduction

Rheumatic heart disease (RHD) affects over 40 million people globally, and accounts for 400.000 deaths annually, most in low- and middle-income countries (1). Rheumatic mitral stenosis (MS) presents slow and progressive narrowing of the mitral orifice and commissural fusion since the initial valvular injury. This lesion results in a stiffer, enlarged and dysfunctional left atrium (LA), increasing the risk for adverse clinical outcomes in these individuals (2).

Percutaneous mitral valvuloplasty (PMV) is the first-line treatment for patients with rheumatic MS and favorable valvular anatomy (3, 4), as it provides immediate hemodynamic alleviation and postpones the need for mitral valve replacement (MVR) (5, 6). PMV is associated with immediate mitral valve area (MVA) enlargement and decreased transmitral gradients, effectively relieving LA pressure overload (7).

Speckle-tracking echocardiography analysis allowed the measurement of left atrial reservoir strain (LASr). LA reservoir function is directly related to its compliance and deformability (8), which are essential in MS to absorb the excessive pressure posed by a narrow mitral orifice. Thus, LASr reflects the LA ability to ease pulmonary hypertension, effectively modulating symptoms in MS patients (9–11). We previously demonstrated that LA reservoir function is a powerful predictor of both new-onset of atrial fibrillation (AF) and overall adverse events (12). However, these analyses were based on isolated measurements of this parameter without considering procedure-related changes (13, 14).

It is known that LASr significantly improves in most patients with rheumatic MS immediately post PMV (15). However, there is a gap in the current knowledge on the impact of this improvement on long-term clinical outcomes. To fill this gap, we sought to analyze the prognostic value of the variation in LASr following PMV in patients with rheumatic MS beyond well-established clinical factors.

## 2 Methods

### 2.1 Study population

A randomly selected subset of patients with rheumatic MS who underwent PMV at a tertiary care center between 2011 and 2023 were recruited and followed. Random subset analysis was performed due to image availability. All patients had a comprehensive transthoracic echocardiogram pre and up to 48 h after the procedure. We excluded patients that required immediate mitral valve replacement (MVR) due to procedural complications. The study was approved by the institutional ethics committee and informed consent was obtained from all individual participants included in the study.

### 2.2 Percutaneous mitral valvuloplasty

Indications for PMV included symptomatic severe mitral stenosis (New York Heart Association class II–IV and MVA assessed by planimetry ≤ 1.5 cm^2^), and favorable mitral valve morphology (4).

### 2.3 Clinical outcomes

The primary outcome was a composite of cardiovascular-related death or the need of MVR. Follow-up was performed at the outpatient clinic every 6–12 months or less, according to symptoms or complications. MVR was indicated after Heart Team evaluation and was driven by the integrated assessment of clinical and echocardiographic findings during follow-up.

### 2.4 Echocardiographic and hemodynamic evaluation

Standard transthoracic two-dimensional and Doppler echocardiographic examinations were performed and analyzed as recommended by the American Society of Echocardiography (3, 16) using commercially available machines.

MVA was measured using 2D direct planimetry by an experienced professional. Peak and mean transmitral diastolic pressure gradients were measured from Doppler profiles in the apical four-chamber view. LV global function was assessed using the biplane Simpson's method. Right ventricular (RV) function was analyzed using tricuspid annular plane systolic excursion (TAPSE), fractional area change (FAC), and peak systolic velocity at the tricuspid annulus using tissue Doppler imaging. Tricuspid regurgitation (TR) and systolic pulmonary artery pressure (SPAP) were evaluated according to the most recent guidelines (3). AF was diagnosed based on a history of permanent AF, supported by a past 12-lead ECG.

LA pressure and compliance, pulmonary arterial pressures, and stroke volume were measured invasively before and after PMV, as detailed elsewhere (17). LA compliance was calculated prior and post PMV with the following formula: Stroke volume (SV)/LA pressure variation during ventricular systole (mL/mmHg). The SV was obtained by dividing the cardiac output, calculated via the Fick method, by heart rate. The LA pressure variation was obtained by subtracting the nadir of the X descent from the peak of the V wave. We performed an average of three sequential beats in patients with normal sinus rhythm (SR) and five in patients with AF, using a scale of 0.5 mmHg/mm.

### 2.5 Assessment of left atrial strain

For measuring LA strain, the LA-focused four-chamber view was acquired using ECG-gated two-dimensional echocardiography (18). Three consecutive cardiac cycles were recorded and averaged, with a frame rate set between 60 and 80 frames per second. Utilizing a single apical view to assess LA strain (as opposed to both apical four- and two-chamber views) is considered acceptable based on a meta-analysis of 30 studies involving 2,038 healthy subjects (19). This meta-analysis provided normative reference values for LA strain during various phases (reservoir, conduit, and contraction). To enhance practicality, the Task Force (18) recommends using LA strain values obtained from an optimized apical four-chamber view that minimizes foreshortening.

**Figure 1:**
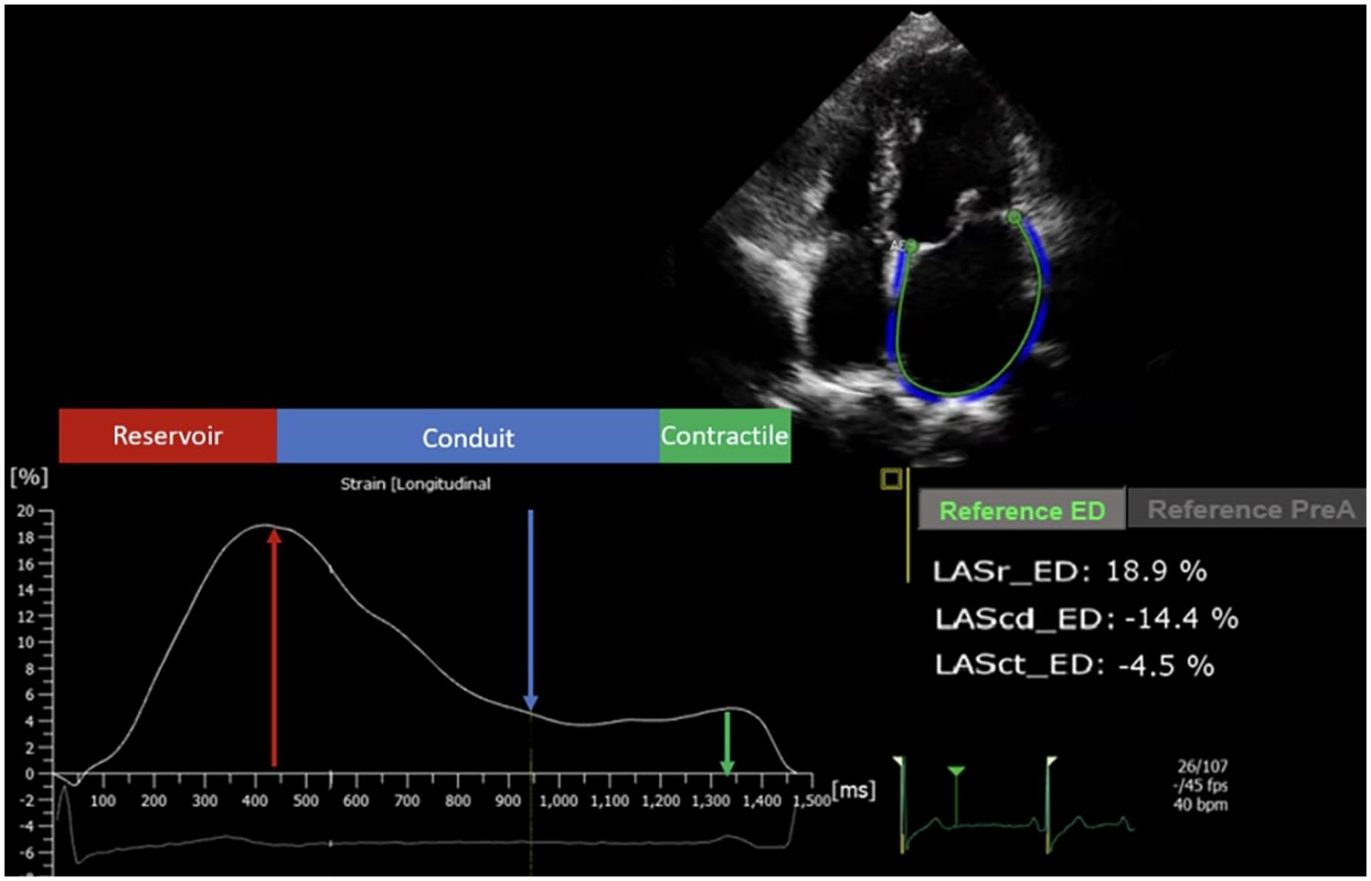
Analysis of left atrial strain using the QRS as a reference. Conduit, conduit phase; Contractile, contractile or pump phase; LAScd_ED, left atrial strain in the conduit phase in end diastole; LASct_ED, left atrial strain in the contractile phase in end diastole; LASr_ED, left atrial strain in the reservoir phase in end diastole. Reservoir: reservoir phase.

LA strain parameters were measured offline using QLAB 15 (Figure 1) with specialized software for LA strain assessment. Strain values were extracted from the strain curves obtained, with peak strain measured during the reservoir (LASr), conduit (LAScd), and contractile (LASct) phases (18).

The rationale for using end diastole (R wave on ECG) as the reference point for measurement lies in its ease of automatic detection by software and its applicability to patients with AF. Furthermore, the reservoir strain measurement, corresponding to the positive peak systolic value of the LA strain curve, can be easily obtained. This is clinically relevant since the reservoir function of the LA is the atrial strain parameter with the most substantial evidence supporting its prognostic utility (8). In addition, LA strain measurements present intra- and inter-observer agreement above 90%, as published elsewhere (12).

### 2.6 Statistical analysis

Categorical variables were expressed as numbers and percentages and were analyzed using chi-squared test. Quantitative variables were expressed as mean ± standard deviation (SD) or median [interquartile range (IQR) 25%–75%] and were compared using 2-tailed unpaired Student's *t*-test or Mann–Whitney test, according to the distribution pattern. To compare paired pre and post PMV measurements, we performed the Wilcoxon signed rank test in quantitative variables and McNemar's test in categorical.

To investigate the prognostic value of the difference in LASr post and pre PMV (ΔLASr), a Cox proportional hazards model was employed. The model was adjusted for clinically relevant or previously validated post PMV prognostic variables (4, 6). Variables that reflected structural changes in the LA were investigated in the Cox model as the variation between post and pre PMV values (ΔLASr, ΔLA pressure and ΔLA compliance). The selection of variables for the final Cox model was determined by the backward elimination method until all variables in the model were statistically significant. A significance level of 5% (α = 0.05) was used to determine statistical significance and confidence intervals were calculated at the 95% level. Statistical analysis was performed using Statistical Package for Social Sciences for Windows, version 28.0 (SPSS Inc., Chicago, IL, USA) and R 4.4.1 (R Foundation for Statistical Computing, Vienna, Austria).

## 3 Results

### 3.1 Clinical characteristics

Among 336 patients who underwent PMV during the study period, 168 were randomly selected to be analyzed, and 163 patients remained after excluding those with unsuccessful PMV. Overall, patients were aged 46 (37–54) years, 82.8% were female, median MVA was 0.97 (0.82–1.13) cm^2^ and median ΔLASr was +3.2 (−0.4 to +6.2). Patients who reached the composite outcome were significantly older (*p* = 0.001), with a higher prevalence of NYHA III or IV (*p* = 0.002), previous valve intervention (surgical commissurotomy or PMV, *p* = 0.010), and moderate or severe TR (*p* = 0.002), and had a lower right ventricular peak systolic tissue velocity (*p* = 0.034). No differences were observed in baseline LA strain parameters before PMV. Baseline characteristics according to the occurrence of the composite outcome are shown in Table 1.

**Table 1:** Baseline characteristics pre PMV stratified by clinical outcomes.

Clinical parameters	No adverse outcome (*n* = 117)	Adverse outcome (*n* = 46)	*p* value
Age (years)	43 (35–52)	51 (44–58)	**0** **.** **001**
Female sex (*n*/%)	95 (81)	40 (87)	0.518
BMI (kg/m^2^)	24.6 (22.5–28.6)	25.2 (23.2–29.3)	0.302
Heart rate (bpm)	70 (60–80)	72 (62–74)	0.850
Atrial Fibrillation (*n*/%)	35 (30)	17 (37)	0.496
NYHA III or IV (*n*/%)	34 (29)	26 (57)	**0**.**002**
Previous intervention (*n*/%)	24 (20.5)	19 (41.3)	**0**.**010**
Hemodynamic measurements			
LA compliance (mL/mmHg)	5.2 (3.2–7.1)	5.4 (3.2–8.9)	0.428
LA pressure (mmHg)	23 (18–28)	21.5 (16–26.9)	0.611
MPAP (mmHg)	30 (23–40)	31 (23.5–38.5)	0.707
Echocardiographic data			
TAPSE (mm)	18 (16–20)	15 (14–20)	0.088
RV FAC (%)	46.1 (36.2–53.2)	45.8 (41.4–51.7)	0.870
RV S’ velocity (cm/s)	10.4 (9.3–11.5)	9.4 (7.8–11.2)	**0**.**034**
LVDd (mm)	48 (44–53)	48 (43–50)	0.268
LVSd (mm)	31 (28–34)	31 (28–34)	0.714
LVEF (%)	63 (59–66)	61 (58–70)	0.594
LA volume index (mL/mm^2^)	55.6 (47.8–71)	58.3 (48.7–75.3)	0.589
Mean gradient (mmHg)	10.5 (8–14)	9.6 (7.1–12.4)	0.088
MVA (cm^2^)	1 (0.8–1.18)	0.92 (0.84–1.1)	0.375
Moderate or severe TR (*n*/%)	14 (12)	16 (35)	**0**.**002**
Mild MR (*n*/%)	91 (78)	40 (87)	0.267
Left atrial strain			
LASr (%)	10.1 (6.2–14.7)	11.1 (6.8–14.6)	0.748
LAScd (%)	4.8 (3.0–7.8)	5.9 (2.9–7.4)	0.878
LASct (%)^a^	6.6 (4.5–9.3)	6.6 (5.6–8.9)	0.960

PMV, percutaneous mitral valvuloplasty; BMI, body-mass-index; LA, left atrium; MPAP, mean pulmonary arterial pressure; TAPSE, tricuspid annular plane systolic excursion; RV FAC, right ventricular fractional area change; RV S’ velocity, right ventricular peak systolic tissue velocity; LVDd, left ventricular diastolic diameter; LVSd, left ventricular systolic diameter; LVEF, left ventricular ejection fraction; MVA, mitral valve area; SPAP, systolic pulmonary artery pressure; TR, tricuspid regurgitation; MR, mitral regurgitation; LASr, left atrial reservoir strain; LAScd, left atrial conduit strain; LASct, left atrial contractile strain.

a LASct was calculated only in patients in normal sinus rhythm, resulting in 82 patients without adverse outcomes and 29 with adverse outcomes.

*p*-values lower than 0.05 (5%) were considered as statistically significant.

### 3.2 Association between parameter changes and clinical outcomes

When comparing pre- and post-PMV parameters as seen in Table 2, patients who reached the composite outcome showed significant improvement in LA compliance (*p* = 0.004), LA pressure (*p* < 0.001), mean pulmonary artery pressure (MPAP, *p* < 0.001), mean gradient (*p* < 0.001), MVA (*p* < 0.001), and LAScd (*p* = 0.006). Similar improvements were also observed in patients who did not experience the outcome. However, patients who did not have the outcome presented an increase in TAPSE (*p* = 0.031), RV FAC (*p* = 0.001), LV diastolic diameter (*p* = 0.005), LV systolic diameter (*p* = 0.018), LASr (*p* < 0.001), and a decrease in LA volume index (*p* < 0.001), while these alterations were not observed in the patients who reached the outcome. The prevalence of moderate MR post PMV was not different between the groups (*p* = 0.39).

**Table 2:** Parameters of the study population pre and post PMV.

Parameters	No adverse outcome (*n* = 117)			Adverse outcome (*n* = 46)		
	Pre PMV	Post PMV	*p* value	Pre PMV	Post PMV	*p* value
Hemodynamic measurements						
LA compliance (mL/mmHg)	5.2 (3.2–7.1)	7.9 (4.5–12.6)	**<0**.**001**	5.4 (3.2–8.9)	7.1 (4.5–11.2)	**0**.**004**
LA pressure (mmHg)	23 (18–28)	15 (11–20)	**<0**.**001**	21.5 (16–26.9)	16.5 (13.3–19.8)	**<0**.**001**
MPAP (mmHg)	30 (23–40)	23 (19–30)	**<0**.**001**	31 (23.5–38.5)	27 (21–32)	**<0**.**001**
Echocardiographic data						
TAPSE (mm)	18 (16–20)	18 (16–21)	**0**.**031**	15 (14–20)	16 (15–19)	0.416
RV FAC (%)	46.1 (36.2–53.2)	50.1 (41.6–55.3)	**0**.**001**	45.8 (41.4–51.7)	50.2 (42.6–54.5)	0.323
RV S’ velocity (cm/s)	10.4 (9.3–11.5)	10.4 (8.9–11.9)	0.852	9.4 (7.8–11.2)	9.6 (8.3–12)	0.145
LVDd (mm)	48 (44–53)	49 (46–53)	**0**.**005**	47.5 (43–50)	47.5 (43–51)	0.790
LVSd (mm)	31 (28–34)	31 (29–36)	**0**.**018**	31 (28–34)	31.5 (28–35)	0.190
LVEF (%)	63 (59–66)	64 (59–67)	0.661	61 (58–70)	61 (56–67)	0.313
LA volume index (mL/mm^2^)	56 (48–71)	53 (43–65)	**<0**.**001**	58.3 (48.7–75.3)	55.6 (49.2–64.5)	0.545
Mean gradient (mmHg)	10.5 (8–14)	5 (4–6)	**<0**.**001**	9.6 (7–12)	5.6 (5–7)	**<0**.**001**
MVA (cm^2^)	1 (0.8–1.2)	1.8 (1.6–1.9)	**<0**.**001**	0.9 (0.8–1.1)	1.5 (1.4–1.7)	**<0**.**001**
Moderate MR (n/%)	–	22 (19)	–	–	12 (26.7)	–
Left atrial strain						
LASr (%)	10.1 (6.2–14.7)	14.1 (8.8–20.1)	**<0**.**001**	11.1 (6.8–14.6)	11 (7.2–17)	0.124
LAScd (%)	4.8 (3.0–7.8)	8.5 (5.3–12.2)	**<0**.**001**	5.9 (2.9–7.4)	7.9 (4.6–10)	**0**.**006**
LASct (%)^a^	6.6 (4.5–9.3)	7.2 (4.4–10.8)	0.472	6.6 (5.6–8.9)	5.7 (3.7–9.8)	0.905

PMV, percutaneous mitral valvuloplasty; LA, left atrium; MPAP, mean pulmonary arterial pressure; TAPSE, tricuspid annular plane systolic excursion; RV FAC, right ventricular fractional area change; RV S’ velocity, right ventricular peak systolic tissue velocity; LVDd, left ventricular diastolic diameter; LVSd, left ventricular systolic diameter; LVEF, left ventricular ejection fraction; MVA, mitral valve area; SPAP, systolic pulmonary artery pressure; MR, mitral regurgitation; LASr, left atrial reservoir strain; LAScd, left atrial conduit strain; LASct, left atrial contractile strain.

a LASct was calculated only in patients in normal sinus rhythm, resulting in 82 patients without adverse outcomes and 29 with adverse outcomes.

*p*-values lower than 0.05 (5%) were considered as statistically significant.

Concerning LASr changes according to ECG rhythm, there were 111 patients in SR and 52 in AF at baseline. Median LASr pre PMV was significantly lower in AF group compared to SR [6% (3.6%–8.7%) vs. 13.2% (8.75–17.1%), *p* < 0.001], and ΔLASr was also significantly lower in AF group [+1.9 (−0.3 to +4.2) vs. +3.7 (−0.4 to +7.4), *p* = 0.049].

### 3.3 Clinical outcomes

Over a median follow-up of 55 months (IQR 27–83), the combined endpoint was reached in 46 patients (28%), being 9 cardiovascular-related deaths and 37 MVR.

In the unadjusted Cox regression model, ΔLASr was significantly associated with the composite outcome [HR: 0.94 (0.89–0.98), *p* = 0.011]. In the final model adjusted for well-established prognostic variables, ΔLASr remained as an independent predictor of the composite outcome [HR: 0.93 (0.87–0.99), *p* = 0.021], as well as age, MVA post PMV, mean gradient post PMV and ΔLA compliance. Results from the Cox models are exhibited in Table 3. The model presented a good discrimination performance (C-statistics of 0.80).

**Table 3:** Cox proportional hazard regression models assessing the predictors of adverse outcomes following PMV.

Parameters	Unadjusted		Multivariable model		Final model	
	HR (95% CI)	*P* value	HR (95% CI)	*P* value	HR (95% CI)	*P* value
ΔLASr	0.94 (0.89–0.98)	0.011	0.92 (0.86–0.98)	0.013	0.93 (0.87–0.99)	0.021
Age	1.06 (1.03–1.09)	<0.001	1.05 (1.01–1.08)	0.011	1.05 (1.02–1.09)	0.002
MVA^a^	0.03 (0.01–0.12)	<0.001	0.16 (0.03–0.82)	0.030	0.16 (0.03–0.73)	0.018
Mean gradient^a^	1.27 (1.11–1.44)	<0.001	1.27 (1.05–1.53)	0.014	1.22 (1.04–1.43)	0.013
ΔLA compliance	0.98 (0.95–1.00)	0.096	0.93 (0.86–0.99)	0.043	0.94 (0.89–0.99)	0.049
ΔLA pressure	0.94 (0.89–0.99)	0.031	1.00 (0.94–1.07)	0.935		
MPAP^a^	1.00 (0.97–1.03)	0.884	0.98 (0.95–1.02)	0.276		
Moderate/severe MR^a^	1.65 (0.85–3.20)	0.140	1.38 (0.57–3.38)	0.476		
Moderate/severe TR^a^	2.41 (1.31–4.43)	0.005	1.23 (0.55–2.79)	0.615		
Atrial Fibrillation	1.76 (0.96–3.21)	0.066	1.15 (0.54–2.42)	0.716		

PMV, percutaneous mitral valvuloplasty; ΔLASr, difference between the absolute values of left atrial reservoir strain post–pre PMV; MVA, mitral valve area; ΔLA compliance, difference between the left atrial compliance post–pre PMV; ΔLA pressure, difference between the left atrial pressure pre–post PMV; MPAP, mean pulmonary arterial pressure; MR, mitral regurgitation; TR, tricuspid regurgitation.

a These measurements in the model were post PMV.

## 4 Discussion

Our study investigated the value of LASr improvement to predict long-term clinical outcomes in patients with rheumatic MS undergoing PMV. Over a median of 55 months of follow-up, our analysis suggests that the higher the increase in LASr post PMV, the lower is the risk for MVR or cardiovascular-related death. Specifically, each unit increase in ΔLASr (post–pre PMV) was associated with a 7% reduction in the risk of adverse outcomes. LASr may be an important prognostic tool in patients with rheumatic MS undergoing PMV.

The other variables that independently predicted adverse outcomes are physiologically plausible. MVA post PMV is well-established as the primary parameter to determine the success of PMV and to determine long-term clinical outcomes in individuals with rheumatic MS (20, 21). Although transmitral gradients are highly dependent of transvalvular flow rate, diastolic filling period, and heart rate (3), they reflect the degree of LA overload and mean gradient post PMV has been shown to predict outcomes in these individuals (20). Athayde et al. demonstrated that improvement in LA compliance post PMV in rheumatic MS is an important predictor of functional status at 6-month follow up (17). We revealed the importance of this improvement in predicting long-term adverse outcomes in these individuals.

Some studies have used speckle-tracking analysis to assess LV function in rheumatic MS. Sengupta et al. demonstrated that LV global longitudinal strain is impaired in rheumatic MS and it significantly improves post PMV (22). In a 4 years follow-up, Barros-Gomes et al. reported that LV global longitudinal strain after PMV is a strong predictor of death and late mitral valve intervention in patients with rheumatic MS (23). Despite the intricate relationship between LA and LV functions, it is well-established that rheumatic MS is essentially an atrial disease with eventual LV dysfunction later in course due to restricted diastolic filling. Therefore, atrial function should be individually assessed.

LASr play an important role in predicting new-onset AF in patients with rheumatic MS (12, 14). Although chronic AF was not an independent predictor of clinical outcomes in our analysis, patients in AF in our cohort had a significantly lower LASr at baseline and had a significantly lower improvement in LASr post PMV compared to patients in SR. As AF disrupts LA structure and mechanics over time, the specific effect of AF on LASr improvement post PMV and over the years of follow-up in rheumatic MS should be addressed in future studies.

Additionally, we previously demonstrated that both LASr and PMV independently predict adverse outcomes in rheumatic MS (12). In 1998, Stefanadis et al. (24) demonstrated an improvement in LA reservoir function post PMV through 2D pressure-area assessment by echocardiography. Pant et al. (25) recently showed that peak LA longitudinal strain improves post PMV in patients with rheumatic MS, and Setouhi et al. (26) showed that LA global longitudinal strain improved immediately after PMV, and the improvement continued at 6 and 12 months post-PMV. However, the impact of LASr variation before and after PMV on long-term clinical outcomes had not yet been explored. Our study is the first to reveal the role of LASr improvement in predicting adverse outcomes post PMV in this patient population.

PMV does not directly address LA fibrosis or intrinsic atrial wall properties. LA strain measurements improve after PMV primarily due to the immediate hemodynamic alleviation with a shift along the pressure-volume curve, as demonstrated by Thomas et al. in 1988 (27). Roslan et al. (15) showed persistent LASr improvement even after 6 months of PMV, revealing the lasting effect of PMV in LA function. As rheumatic MS is a complex condition that encompasses both a biological and a hemodynamic component, a comprehensive assessment of left atrial function, morphology and properties is essential to improve patient management.

The study by Roslan et al. investigated 136 patients with rheumatic MS undergoing PMV and found that LASr was the only analyzed strain parameter with significant immediate and 6 months improvement post PMV in rheumatic MS patients (15). Despite that, they did not find an association between LASr and need for reintervention in 6 months. The design of the study conducted by Roslan et al. was important to track the changes in echocardiographic and strain parameters immediately after and 6 months post PMV. However, the short timeframe of 6 months may not have been sufficient to reveal the difference in adverse outcome rates associated with the comparison of LASr post and pre PMV. Our study included more patients (163) in a time-to-event analysis, with a significantly longer follow-up of the patients (median of 55 months), revealing the prognostic importance of the improvement of LASr in PMV in this population.

LA strain is a useful and sensitive tool to identify LA dysfunction caused by rheumatic MS. LASr effectively reflects LA reservoir function, being correlated with LA fibrosis and stiffness, which are associated with adverse outcomes in this population. Our cohort highlights the importance of the change in LASr pre and post PMV as a valuable long-term prognostic tool in rheumatic MS. Integrating this technique with the standard echocardiographic and clinical practice may provide key insights for guiding patient care.

Our study has some limitations. First, this is a single-center study, and the results may not be generalizable to the overall population. Despite that, our cohort exhibits characteristics consistent with broader MS populations, with a clear female predominance in the fourth or fifth decades of life. Second, the images in which the atrial strain was measured were not acquired specifically for performing LA strain in terms of orientation, depth, gain, and frame rate. Additionally, we did not categorize AF into paroxysmal, permanent, and longstanding, factors known to impact LASr. Our study also did not assess functional status and hospitalization rates, outcomes that are important in rheumatic MS both for clinical management and for indication of intervention.

In conclusion, the magnitude of increase in LASr following PMV emerged as a strong predictor of clinical outcomes in patients with rheumatic MS. Our findings indicate that a greater ΔLASr is associated with a reduced risk of adverse clinical outcomes, highlighting its potential in risk stratification following PMV.

## Data Availability

The raw data supporting the conclusions of this article will be made available by the authors, without undue reservation.

## In Memoriam

This work is dedicated to Dr. Robert A. Levine, a mentor, colleague, and friend, whose vision and mentorship shaped our understanding of cardiovascular disease and whose memory continues to inspire our efforts to advance care for patients suffering from rheumatic heart disease worldwide.

## References

[1] Roth GA Mensah GA Johnson CO Addolorato G Ammirati E Baddour LM Global burden of cardiovascular diseases and risk factors, 1990–2019: update from the GBD 2019 study. J Am Coll Cardiol. (2020) 76:2982–3021. 10.1016/j.jacc.2020.11.010 33309175 PMC7755038

[2] Nunes MCP Tan TC Elmariah S Lodi-Junqueira L Nascimento BR do Lago R Net atrioventricular compliance is an independent predictor of cardiovascular death in mitral stenosis. Heart. (2017) 103:1891–8. 10.1136/heartjnl-2016-310955 28780580

[3] Otto CM Nishimura RA Bonow RO Carabello BA Erwin JP Gentile F 2020 ACC/AHA guideline for the management of patients with valvular heart disease: a report of the American college of cardiology/American heart association joint committee on clinical practice guidelines. Circulation. (2021) 143:e72–e227. 10.1161/CIR.0000000000000923 33332150

[4] Nunes MCP Tan TC Elmariah S do Lago R Margey R Cruz-Gonzalez I The echo score revisited. Circulation. (2014) 129:886–95. 10.1161/CIRCULATIONAHA.113.001252 24281331

[5] Nobuyoshi M Arita T Shirai S Hamasaki N Yokoi H Iwabuchi M Percutaneous balloon mitral valvuloplasty. Circulation. (2009) 119:e211–9. 10.1161/CIRCULATIONAHA.108.792952 19106383

[6] Meneguz-Moreno RA Ribamar Costa J Gomes NL Braga SLN Ramos AIO Meneghelo Z Very long term follow-up after percutaneous balloon mitral valvuloplasty. JACC Cardiovasc Interv. (2018) 11:1945–52. 10.1016/j.jcin.2018.05.039 30077684

[7] Arora R Nair M Kalra GS Nigam M Khalilullah M. Immediate and long-term results of balloon and surgical closed mitral valvotomy: a randomized comparative study. Am Heart J. (1993) 125:1091–4. 10.1016/0002-8703(93)90118-S 8465732

[8] Thomas L Muraru D Popescu BA Sitges M Rosca M Pedrizzetti G Evaluation of left atrial size and function: relevance for clinical practice. J Am Soc Echocardiogr. (2020) 33:934–52. 10.1016/j.echo.2020.03.021 32762920

[9] Bouchahda N Kallala MY Zemni I Ben Messaoud M Boussaada M Hasnaoui T Left atrium reservoir function is central in patients with rheumatic mitral stenosis. Int J Cardiovasc Imaging. (2022) 38:1257–66. 10.1007/s10554-021-02509-4 34971418

[10] Mahfouz RA Gouda M Abdelhamed M. Relation between left atrial strain and exercise tolerance in patients with mild mitral stenosis: an insight from 2D speckle-tracking echocardiography. Echocardiography. (2020) 37:1406–12. 10.1111/echo.14818 32777140

[11] Chien C-Y Chen C-W Lin T-K Lin Y Lin J-W Li Y-D Atrial deformation correlated with functional capacity in mitral stenosis patients. Echocardiography. (2018) 35:190–5. 10.1111/echo.13770 29226357

[12] Figueiredo F de A Esteves WAM Hung J Gomes NFA Taconeli CA Pantaleão AN Left atrial function in patients with rheumatic mitral stenosis: addressing prognostic insights beyond atrial fibrillation prediction. Eur Heart J Imaging Methods Pract. (2024) 2:qyae067. 10.1093/ehjimp/qyae067 39224865 PMC11367946

[13] Kuppahally SS Akoum N Burgon NS Badger TJ Kholmovski EG Vijayakumar S Left atrial strain and strain rate in patients with paroxysmal and persistent atrial fibrillation: relationship to left atrial structural remodeling detected by delayed-enhancement MRI. Circ Cardiovasc Imaging. (2010) 3:231–9. 10.1161/CIRCIMAGING.109.865683 20133512

[14] Stassen J Butcher SC Namazi F Ajmone Marsan N Bax JJ Delgado V. Left atrial deformation imaging and atrial fibrillation in patients with rheumatic mitral stenosis. J Am Soc Echocardiogr. (2022) 35:486–494.e2. 10.1016/j.echo.2021.12.010 34954048

[15] Roslan A Aris FA Sin TY Ashari A Shaparudin AA Shah WFWR Comprehensive echocardiographic and speckle tracking strain analysis in rheumatic mitral stenosis patients before and after transvenous mitral commissurotomy. Int J Cardiovasc Imaging. (2022) 38:1307–16. 10.1007/s10554-021-02518-3 34978670

[16] Lang RM Badano LP Mor-Avi V Afilalo J Armstrong A Ernande L Recommendations for cardiac chamber quantification by echocardiography in adults: an update from the American society of echocardiography and the European association of cardiovascular imaging. J Am Soc Echocardiogr. (2015) 28:1–39.e14. 10.1016/j.echo.2014.10.003 25559473

[17] Athayde GRS Nascimento BR Elmariah S Lodi-Junqueira L Soares JR Saad GP Impact of left atrial compliance improvement on functional status after percutaneous mitral valvuloplasty. Catheter Cardiovasc Interv. (2019) 93:156–63. 10.1002/ccd.27831 30244517 PMC8272835

[18] Badano LP Kolias TJ Muraru D Abraham TP Aurigemma G Edvardsen T Standardization of left atrial, right ventricular, and right atrial deformation imaging using two-dimensional speckle tracking echocardiography: a consensus document of the EACVI/ASE/Industry Task Force to standardize deformation imaging. Eur Heart J Cardiovasc Imaging. (2018) 19:591–600. 10.1093/ehjci/jey042 29596561

[19] Pathan F D’Elia N Nolan MT Marwick TH Negishi K. Normal ranges of left atrial strain by speckle-tracking echocardiography: a systematic review and meta-analysis. J Am Soc Echocardiogr. (2017) 30:59–70.e8. 10.1016/j.echo.2016.09.007 28341032

[20] Mohanan Nair KK Valaparambil A Sasidharan B Ganapathi S Gopalakrishnan A Namboodiri N Immediate and late clinical outcomes of balloon mitral valvotomy based on immediate postballoon mitral valvotomy mitral valve area & percentage gain in mitral valve area—a tertiary centre study. Indian Heart J. (2018) 70:S338–46. 10.1016/j.ihj.2018.09.005 30595286 PMC6309712

[21] Hernandez R Bañuelos C Alfonso F Goicolea J Fernández-Ortiz A Escaned J Long-term clinical and echocardiographic follow-up after percutaneous mitral valvuloplasty with the inoue balloon. Circulation. (1999) 99:1580–6. 10.1161/01.CIR.99.12.1580 10096934

[22] Sengupta SP Amaki M Bansal M Fulwani M Washimkar S Hofstra L Effects of percutaneous balloon mitral valvuloplasty on left ventricular deformation in patients with isolated severe mitral stenosis: a speckle-tracking strain echocardiographic study. J Am Soc Echocardiogr. (2014) 27:639–47. 10.1016/j.echo.2014.01.024 24637055

[23] Barros-Gomes S Eleid MF Dahl JS Pislaru C Nishimura RA Pellikka PA Predicting outcomes after percutaneous mitral balloon valvotomy: the impact of left ventricular strain imaging. Eur Heart J Cardiovasc Imaging. (2017) 18:763–71. 10.1093/ehjci/jew160 27502294

[24] Stefanadis C Dernellis J Stratos C Tsiamis E Vlachopoulos C Toutouzas K Effects of balloon mitral valvuloplasty on left atrial function in mitral stenosis as assessed by pressure-area relation. J Am Coll Cardiol. (1998) 32:159–68. 10.1016/s0735-1097(98)00178-8 9669265

[25] Pant BP Walse R Sivadasapillai H Ganapathi S Valaparambil A. Left atrial strain as a surrogate parameter for successful percutaneous ballon mitral valvotomy? Acta Cardiol. (2025) 80:274–82. 10.1080/00015385.2025.2484506 40173301

[26] Setouhi A Boshra H Askalany H Farrag HMA. Immediate, short-term, and long-term effects of balloon mitral valvuloplasty on the left atrial global longitudinal strain and its correlation to the outcomes in patients with severe rheumatic mitral stenosis. Egypt Heart J. (2023) 75:98. 10.1186/s43044-023-00425-7 38038813 PMC10692051

[27] Thomas JD Wilkins GT Choong CY Abascal VM Palacios IF Block PC Inaccuracy of mitral pressure half-time immediately after percutaneous mitral valvotomy. Dependence on transmitral gradient and left atrial and ventricular compliance. Circulation. (1988) 78:980–93. 10.1161/01.cir.78.4.980 3168200

